# The burden of diarrhoea, shigellosis, and cholera in North Jakarta, Indonesia: findings from 24 months surveillance

**DOI:** 10.1186/1471-2334-5-89

**Published:** 2005-10-20

**Authors:** Magdarina D Agtini, Rooswanti Soeharno, Murad Lesmana, Narain H Punjabi, Cyrus Simanjuntak, Ferry Wangsasaputra, Dazwir Nurdin, Sri Pandam Pulungsih, Ainur Rofiq, Hari Santoso, H Pujarwoto, Agus Sjahrurachman, Pratiwi Sudarmono, Lorenz von Seidlein, Jacqueline L Deen, Mohammad Ali, Hyejon Lee, Deok Ryun Kim, Oakpil Han, Jin Kyung Park, Agus Suwandono, Ingerani, Buhari A Oyofo, James R Campbell, H James Beecham, Andrew L Corwin, John D Clemens

**Affiliations:** 1 National Institute of Health Research and Development, Jakarta Indonesia, Ministry of Health, Jakarta, Indonesia; 2 United States Navy Medical Research Unit 2, Jakarta, Indonesia; 3 Infectious Disease Hospital Sulianti Saroso, Ministry of Health, Jakarta, Indonesia; 4 Communicable Disease Control and Environmental Health, Ministry of Health, Jakarta, Indonesia; 5 Department of Microbiology, University of Indonesia, Jakarta, Indonesia; 6 International Vaccine Institute, Seoul, Korea; 7 National Institute of Child Health and Human Development, Bethesda, Maryland, USA

## Abstract

**Background:**

In preparation of vaccines trials to estimate protection against shigellosis and cholera we conducted a two-year community-based surveillance study in an impoverished area of North Jakarta which provided updated information on the disease burden in the area.

**Methods:**

We conducted a two-year community-based surveillance study from August 2001 to July 2003 in an impoverished area of North Jakarta to assess the burden of diarrhoea, shigellosis, and cholera. At participating health care providers, a case report form was completed and stool sample collected from cases presenting with diarrhoea.

**Results:**

Infants had the highest incidences of diarrhoea (759/1 000/year) and cholera (4/1 000/year). Diarrhea incidence was significantly higher in boys under 5 years (387/1 000/year) than girls under 5 years (309/1 000/year; p < 0.001). Children aged 1 to 2 years had the highest incidence of shigellosis (32/1 000/year). *Shigella flexneri *was the most common *Shigella *species isolated and 73% to 95% of these isolates were resistant to ampicillin, trimethoprim-sulfamethoxazole, chloramphenicol and tetracycline but remain susceptible to nalidixic acid, ciprofloxacin, and ceftriaxone. We found an overall incidence of cholera of 0.5/1 000/year. Cholera was most common in children, with the highest incidence at 4/1 000/year in those less than 1 year of age. Of the 154 *V. cholerae *O1 isolates, 89 (58%) were of the El Tor Ogawa serotype and 65 (42%) were El Tor Inaba. Thirty-four percent of patients with cholera were intravenously rehydrated and 22% required hospitalization. *V. parahaemolyticus *infections were detected sporadically but increased from July 2002 onwards.

**Conclusion:**

Diarrhoea causes a heavy public health burden in Jakarta particularly in young children. The impact of shigellosis is exacerbated by the threat of antimicrobial resistance, whereas that of cholera is aggravated by its severe manifestations.

## Introduction

Diarrhoeal diseases remain a major cause of morbidity and mortality in all age groups in impoverished areas of South East Asia [1-5]. In Indonesia, diarrhoea is the third leading cause of overall morbidity and the leading cause of infant mortality [6,7]. Earlier studies extensively explored the causative organisms of diarrhoea in the slums of Indonesia's capital, Jakarta [8-11]. In a study conducted from 1997 to 1999, *Shigella flexneri *was found to be the most frequently isolated organism from diarrhoea patients in a community setting in Jakarta [9]. Similarly, *S. flexneri *was the most common organism isolated in eight hospitals throughout the Indonesian archipelago between 1999 and 2000 [10]. Cholera is another persistent problem in Indonesia. In studies of diarrhoeal outbreaks throughout Indonesia from 1993 to 1999, *Vibrio cholerae *O1 was isolated from 8% to 11% of stool samples collected [8,12,13].

To plan trials for the evaluation of vaccine candidates it is essential to understand the disease incidence in the study area. In preparation of vaccines trials to estimate protection against shigellosis and cholera we conducted a two-year community-based surveillance study in an impoverished area of North Jakarta. The experience gained during the surveillance will be useful for future vaccine trials as well as for the understanding of endemic disease rates in urban slums in the region.

## Methods

### Study area and population

North Jakarta, one of five municipal administrative areas of the city, is 154 km^2^, had a population of 1 435 207 in 2000, and an estimated population density of 9 314 individuals/square kilometre. The average annual income per person in this congested urban site was US$ 689. – in 2000 [14]. Water supply and sanitation are inadequate. Many homes are temporary structures without running water and more than a third of households have no access to tap water. Two adjacent districts (*kecamatans*) in North Jakarta, Tanjung Priok and Koja, were selected for the study based on expected high incidence of the target diseases, accessibility and previous research experience in the area [15,16]. Residents of the socio-economically better-off areas of the study site did not participate in the study due to their known preference for private health care. The total population enumerated by a study census in 2001 was 160 261 individuals of whom 15 741 (10%) were less than 60 months of age [17]. The local climate has 2 distinctive seasons: a rainy season (December–April) and a dry season (May–November)[18].

### Health care system

In the Indonesian public sector, the first-level health care facility is the public health centre, locally known as the *puskesmas*. North Jakarta has 54 public health centres, 22 of which are located in Tanjung Priok and Koja. More severe conditions are referred to government hospitals. The Infectious Disease Hospital and Koja Hospital are the main government referral hospitals in North Jakarta. In 2001, there were 314 private practitioners, 36 polyclinics, 32 maternal clinics, and 29 mostly very small private hospitals in the study area.

### Study surveillance procedures

The surveillance was conducted from August 2001 to July 2003. The study was designed to detect all patients residing in the two study districts presenting with diarrhoea at participating health care providers: public health centres in the study area, the Infectious Disease Hospital, and Koja Hospital. A 2001 survey in the study area asked 8 074 households that had diarrhoea cases in the preceding four weeks where care was sought [17], and 39% reported they would use a treatment provider taking part in the surveillance. Based on these findings, we estimate that 61% of diarrhoea episodes were not captured by our surveillance system.

In the present study, we invited consenting patients residing in the study area of all age groups presenting to a participating health care provider with loose bowel movement to join the study. Diarrhoea was defined as passage of three or more loose stools in 24 hours or one or more loose stool with visible blood. A new diarrhoea episode was defined if the diarrhoea definition was met after three of more days free of diarrhoea or dysentery. Shigellosis was defined as diarrhoea with isolation of *Shigella *species from a stool sample. Cholera was defined as diarrhoea with isolation of *V. cholerae *O1 or O139 from a stool sample. Fever was defined as axillary temperature of 37.5°C or higher.

Study personnel were trained in study procedures. For every patient presenting with diarrhoea agreeing to take part in the study, a case report form was completed describing address, demographics, medical history, physical examination results, and the treatment plan including the prescription of antibiotics. Patients who did not consent or had no diarrhea were excluded. Three rectal swabs were obtained from all study participants. Treatment was provided to patients in accordance with national guidelines and the treatment plan including hospital referral was recorded. The study did not include follow-up visits of patients.

For isolation of *Shigella *species, one rectal swab was placed in buffered glycerol saline (BGS) and another in potassium buffered saline. Both swabs were refrigerated and transported in a cool box to the central laboratory by motorcycle and plated on the same day they were obtained.

The specimens in BGS were plated on MacConkey agar and *Salmonella-Shigella *agar. Biochemical reactions of colonies were evaluated in Kligler's iron agar and motility indole ornithine medium. Colonies were serologically confirmed by slide agglutination with appropriate group-specific polyvalent antiserum, followed by type-specific monovalent antisera. Standardized, commercial antisera (Denka Seiken Co., Ltd. 3-4-2, Nihonbashi, Kayaba-Cho, Chuo-Ku, Tokyo 103-0025, Japan) were used for identification. In cases where no agglutination occurred with live bacteria, the test was repeated with boiled suspensions of bacteria.

For isolation of *Vibrios*, one rectal swab was placed in Cary-Blair transport medium, kept at room temperature, and transported to the central laboratory by motorcycle and plated on the same day that were obtained. From the Cary-Blair media, the specimens were plated directly onto thiosulfate citrate bile salt sucrose (TCBS) agar (Eiken Chemical Company, Tokyo, Japan) and also plated onto TCBS after enrichment in alkaline peptone water for 6 and 20 hours (pH 8.6, 37°C). After incubation overnight, suspected colonies on the TCBS plates were tested biochemically and agglutinated with polyvalent, Ogawa, and Inaba antisera (Difco Laboratories, Detroit, Michigan). Non-agglutinating strains were tested with antiserum to *V. cholerae *O139 strain. Identification of *Vibrio parahaemolyticus *was done according to standard methods [19].

Antimicrobial susceptibility testing of *Shigella *and *V. cholerae *isolates against ampicillin, trimethoprim-sulfamethoxazole, chloramphenicol, tetracycline, cefotaxime, ceftriaxone, ciprofloxacin, and nalidixic acid was performed using standard antibiotic discs (Becton, Dickinson and Co., Sparks, MD). Isolates were assessed as being resistant, intermediate or susceptible according to standard cut-off zone sizes [20]. A control strain, *Escherichia coli *ATCC 25922, was included in the test.

### Data management and analysis

All case report forms were double entered into custom-made data entry programs using FoxPro software (Microsoft, Redmond, WA). The data management programs include error as well as consistency check programs. SAS software (SAS Institute Inc., Cary, NC) was used for statistical analyses. Because ascertainment of cases was passive, we did not know how many of the people included in the study stayed in the study area. We estimated person-years at risk assuming the study population stayed in the study area until the end of the study period. We used the age-specific number of disease episodes in residents of the study area as the numerator. Incidence was calculated based on the population residing in the catchment area in 2001 as the denominator. A test for trend (chi square) was applied to assess the statistical significance of increasing incidence rates of shigellosis with increasing age (after age 30 years). For nonparametric data, Wilcoxon rank sum test was used for the comparison of two groups. Chi square test was used for the analysis of binary data. Student's t test or chi square test was used, as appropriate, to compare clinical features of cases by etiologic organism.

### Ethics

After the project's purpose was explained, patients, or in the case of minors, their parent or guardian, gave verbal consent prior to participation in the study. Participation consisted of providing a stool specimen and the information required to complete the case report forms. The study was approved by the Ethics Committee of the Ministry of Health, Indonesia; the Institutional Review Board, National Institute of Health Research and Development, Ministry of Health, Jakarta, Indonesia; the Institutional Review Board, United States Naval Medical Research Unit No 2; and from the Secretariat Committee on Research Involving Human Subjects, World Health Organization, Geneva, Switzerland.

## Results

### Diarrhoea

**Figure 1:**
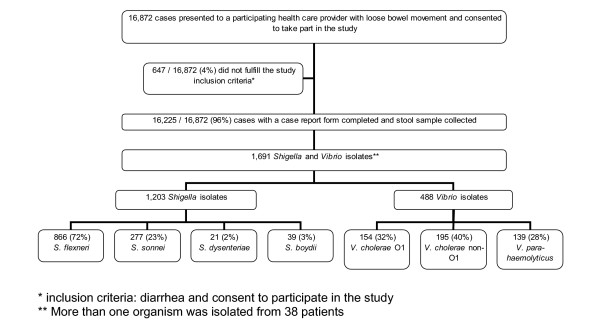
Flow of diarrhoea patients.

**Figure 2:**
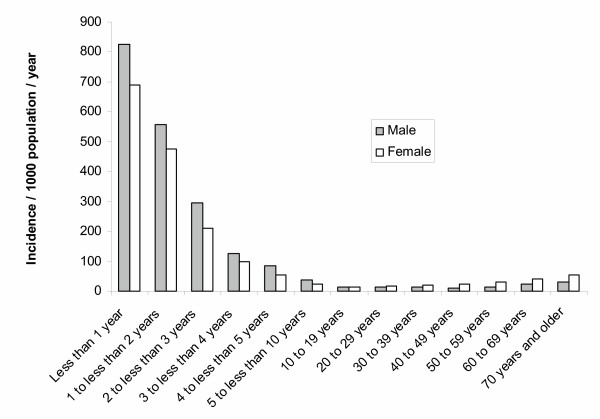
Incidence of diarrhoea by sex and age group, North Jakarta, August 2001 to July 2003.

**Figure 3:**
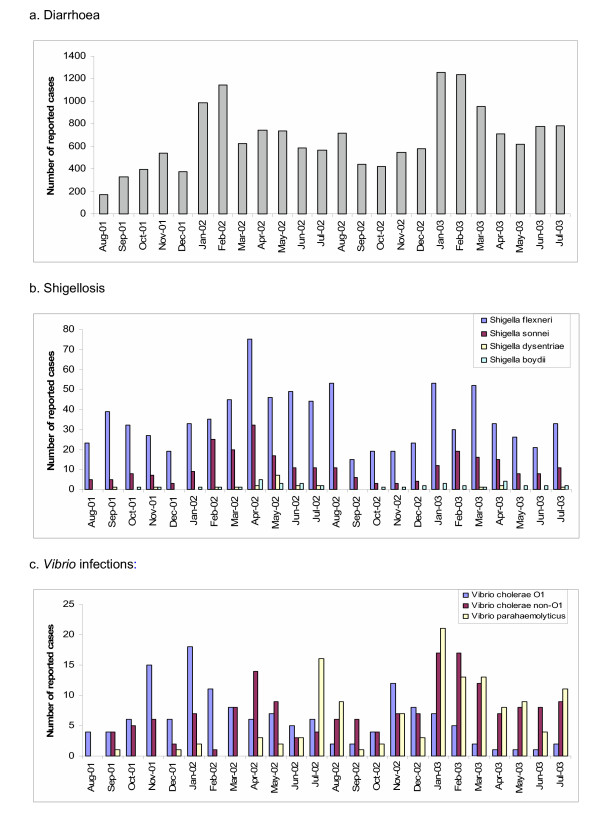
Seasonality of diarrhoea, shigellosis and *Vibrio *infections, North Jakarta a. Diarrhoea, b. *Shigellosis*, c. Vibrio infections.

From August 2001 through July 2003, 16 872 patients presented to one of the participating treatment centres with loose bowel movements and agreed to take part in the study (Figure 1). 647 (4%) who did not fulfil the study criteria (consent and diarrhea) were excluded and 16 225 (96%) diarrhoea cases were analyzed. In all, 1 203 *Shigella *and 488 *Vibrio *organisms were isolated from the stool samples. More than one organism was isolated from 38 patients. We found an overall incidence of diarrhoea of 50 per 1 000 population per year. The highest diarrhoea incidence was in infants less than 1 year of age at 759 cases/1 000/year (Figure 2). Sixty-eight percent (10 998/16 225) of diarrhoea cases were in children less than 5 years of age (incidence of 349/1 000/year). Diarrhea incidence was significantly higher in boys under 5 years (387/1 000/year) than girls under 5 years (309/1 000/year; p < 0.001). In contrast the reported diarrhea incidence in females 5 years and older (21/1 000/year) was significantly higher than in males 5 years or older (16/1 000/year; p < 0.001). Diarrhoea cases had a clear seasonality. During the first three months of the year (January, February, March) 1578/16225 (38%) of diarrhea episodes were captured and during the remainig 9 months 10 026 (62%) of the diarrhea episodes were captured (p < 0.001; Figure 3).

### Shigellosis

**Figure 4:**
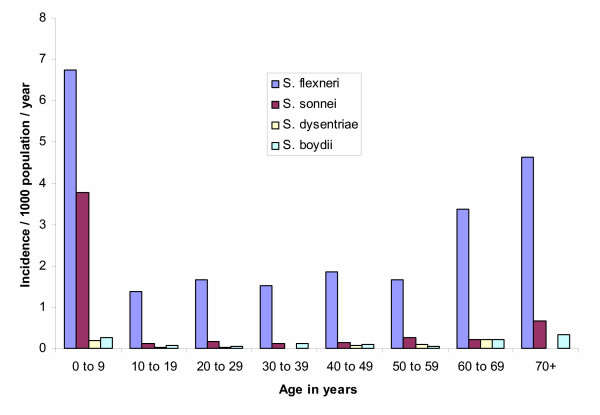
Incidence of shigellosis by *Shigella *species and age group infected, North Jakarta, August 2001 through July 2003.

We found an overall incidence of shigellosis of 4/1 000/year. Shigellosis was most common among children and the elderly (Figure 4), with the highest incidence at 32/1 000/year in those 1 to 2 years of age. Of the 1 203 *Shigella *isolates, 866 (72%) were *S. flexneri*, 277 (23%) were *S. sonnei*, 21 (2%) were *S. dysenteriae *(none of which were *S. dysenteriae *type 1), and 39 (3%) were *S. boydii *(Figure 1). Incidence by type of specie was 2.6/1 000/year for *S. flexneri*, 0.8 for *S. sonnei*, 0.1 for *S. dysenteriae*, and 0.1 for *S. boydii*. The most frequently encountered *S. flexneri *serotypes were 2a and 3a, followed by 1b and 1c (Table 1). Two percent of the *S. flexneri *isolates could not be typed with commercially available antisera. We evaluated the antimicrobial resistance pattern of the *Shigella *isolates by species. In all, 73% to 95% of *S. flexneri *isolates were resistant to ampicillin, trimethoprim-sulfamethoxazole, chloramphenicol, and tetracycline. None showed resistance to ciprofloxacin and only a single isolate (of *S. flexneri*) was not susceptible to ceftriaxone and nalidixic acid. Significantly more shigellosis cases were detected between February and April (418/1772; 36%) during the rainy months compared to the remaining 9 months of the year (754/1172; 64%; p < 0.001; Figure 3).

**Table 1:** *Shigella flexneri *serotypes detected in North Jakarta, August 2001 to July 2003

**Serotypes**	**Number**	**(%)**
1a	63	7
1b	105	12
1c	103	12
2a	297	34
2b	5	1
3a	142	16
3b	10	1
4	12	1
4a	53	6
4b	1	<1
4X	11	1
5a	2	<1
6	38	4
X	2	<1
Y	5	1
Non-typeable	17	2

**Total**	**866**	**100**

### Cholera and other vibriosis

**Figure 5:**
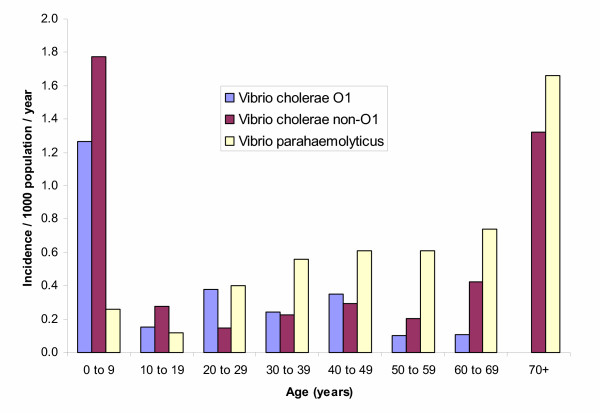
Incidence of *Vibrio cholerae *and *Vibrio parahaemolyticus *by age group, North Jakarta, August 2001 through July 2003.

We found an overall incidence of cholera of 0.5/1 000/year. Cholera was most common in children (Figure 5), with the highest incidence at 4/1 000/year in those less than 1 year of age. Of the 154 *V. cholerae *O1 isolates, 89 (58%) were of the El Tor Ogawa serotype and 65 (42%) were El Tor Inaba. *V. cholerae *O139 was not isolated during the two-year study period. The majority of isolates (>90%) remain susceptible to the first-line antimicrobial agents, trimethoprim-sulfamethoxazole and tetracycline as well as nalidixic acid, ciprofloxacin and chloramphenicol. Cholera peaked in December. Significantly more cases were detected between December and March (162/443; 37%) during the cooler months compared to the remaining 9 months of the year (281/443; 37%; p < 0.001; Figure 3).

During the surveillance, other *Vibrio *organisms were also detected. The incidence of diarrhoea due to *V. cholerae *non-O1 was 0.5/1 000/year and that due to *V. parahaemolyticus *was 0.4/1 000/year. *V. cholerae *non-O1 diarrhoea was most common among children and the elderly, whereas the incidence of *V. parahaemolyticus *diarrhoea gradually increased with age (Figure 5). Seasonality was not clear for diarrhoea associated with *V. cholerae *non-O1 and *V. parahaemolyticus *(Figure 3). Initially, *V. parahaemolyticus *infections were detected sporadically but increased from July 2002 onwards.

### Clinical features and management

We assessed clinical features and management of the diarrhoea cases and separately, those from whom *Shigella *and *Vibrio *strains were isolated, excluding the 38 cases from whom more than one organism was isolated (Table 4). The mean and median ages of cases with diarrhoea due to *V. parahaemolyticus *were significantly higher than those of cases caused by other *Vibrio *or *Shigella *species (p < 0.001). Only 10 to 25% of shigellosis cases, depending on the specie, reported blood in the stool. Nearly all patients (range 90 to 100%) were prescribed antibiotics. Ten percent of patients with cholera were severely dehydrated, 34% were intravenously rehydrated, and 22% were referred for hospital admission. Compared to cholera cases, all other diarrhoea cases were less likely to have severe dehydration (P value < 0.001), rehydrated with intravenous fluids (P value < 0.001), or be hospitalized (P value = 0.001).

## Discussion

This is the first community-based surveillance of diarrhoea in Jakarta and our findings underscore its public health significance especially amongst children less than 60 months of age. By utilizing passive surveillance, that is detecting patients coming to health centres for treatment, we found an annual incidence of diarrhoea requiring medical attention of 349 per 1 000 children and 759 per 1000 infants.

The study was designed in preparation of vaccine trials trying to find an appropriate population for the evaluation of vaccine candidates. The residents of two kecamatans, Tanjung Priok and Koja were selected as study population for the study based on their accessibility and the relative high incidence of the target diseases. We have found that in this population only 39% of diarrhea episodes overall would trigger a visit to a health care centre. Taking into account the lack of sensitivity of the classic culture methods used in the study it is reasonable to assume that the true disease incidence could be several fold higher than our estimates. Our study population is likely to be representative of the less affluent segment of the population of Jakarta which depends on the public health care system. However the study population was not chosen because it is representative for the population of Jakarta. More affluent households in North Jakarta may prefer to make use of the private sector and were not captured by the study. The incidence of the target diseases estimated by our study may differ in the more affluent population.

The incidence of shigellosis was highest in children between 1 and 2 years of age, consistent with previous data indicating that maternal antibodies and breast-feeding may protect newborns and infants [21-23]. We found increasing rates of shigellosis in those 70 years of age and older, which could be related to waning of immunity. *S. flexneri *was the most frequently detected Shigella species and four *S. flexneri *serotypes (2a, 3a, 1b, 1c) made up 74% of the *S. flexneri *isolates. This finding is consistent with those from a previous report that suggested that the most frequent *S. flexneri *serotypes are 2a, 1b, and 3b [24].

The highest number of diarrhea cases was detected each year during the rainy season between January and April when North Jakarta is particularly flood prone. The number of detected cholera cases peaked each year in January. Relatively few *V. parahaemolyticus *strains were detected during the first year of the study which became the dominant *Vibrio *species towards the end of the study. The increase in *V. parahaemolyticus *detection may well have been related to the pandemic spread of the *V. parahaemolyticus *serovar O3:K6 though the serovar of the strains has yet to be determined. The detection of all *Shigella* strains coincided with the rainy season (January through April) and had a distinct peak in April of the first year of surveillance.

The greatest burden of cholera was in children during the first year of life. Previously, cholera was believed to occur infrequently below 2 years of age [25] but more recent studies have found cholera to be a significant problem in young children [26,27]. We found significant numbers of diarrhoea cases due to other *Vibrio *species. These diarrhoea episodes were more likely to occur in adults than in children as previously reported [8,28]. *V. parahaemolyticus *infections are transmitted through unsafe foods, such as raw or undercooked seafood, which are more likely to be consumed by adults [8]. *V. cholerae *non-O1 and *V. parahaemolyticus *were associated with milder illness than *V. cholerae *O1.

The large majority of study patients received rehydration fluids (mostly orally) in accord with international guidelines. Only 4% of diarrhoea patients reported taking antibiotics prior to presentation which is surprising considering the ubiquitous presence of drug vendors in the study area. Resistance of *Shigella *to quinolones is not yet a major problem in the area but resistance to the more commonly used first-line antimicrobials is disturbing. Ampicillin, tetracycline, chloramphenicol, and trimethoprim-sulfamethoxazole are of little use for treatment of *S. flexneri *in the study area. Antimicrobial resistance of *V. cholera*e is not yet a major problem in Jakarta. However the surprisingly high percentage of diarrhea patients who were prescribed antibiotics is worrying as the drug pressure is likely to select resistant strains and is unlikely to benefit many the patients.

## Conclusion

In conclusion, diarrhoea causes a heavy public health burden in Jakarta. The impact of shigellosis is exacerbated by the threat of antimicrobial resistance, whereas that of cholera is aggravated by its severe manifestations.

## Competing interests

No author has political, personal, religious, academic, intellectual, commercial or any other interests that could lead to a conflict of interest. The views expressed in this paper are those of the authors and do not necessarily represent the views of the United States Department of the Navy or Department of Defence.

## Authors' contributions

The principal investigators were CS during the first 12 months of the study and MA during the remainder of the study. NP and RS coordinated the local study activities during the first and second year respectively. ML, DN, BO, JRC, HJB, and ALC were responsible for the microbiological aspects of the study at United States Navy Medical Research Unit 2; ASj and PS coordinated the microbiological component at the University of Indonesia. SPP and HS coordinated the study activities in the hospitals. AR, AS, and I served as liaison with the Ministry of Health, the communities and the investigators. LvS coordinated the epidemiologic aspects of the overall project. DRK and MA developed and managed the database which was administered and supervised by FW, DRK, OH and JKP managed and analyzed the data. JLD wrote the first draft of the manuscript, JC conceived the project, attracted funding and oversaw all stages of the project. All authors contributed to the writing of the final version of this paper.

**Table 2:** Clinical features and management of diarrhoea cases, by organism isolated (excluding 38 cases with mixed infections)

	**Diarrhoea cases N = 16,187**	***Shigella***				***Vibrio***		
		
		***flexneri *N = 844**	***sonnei *N = 269**	***dysenteriae *N = 21**	***boydii *N = 38**	***cholerae *O1 N = 143**	***cholerae *non-O1 N = 171**	***parahaemolyticus *N = 129**
Mean (median) age in years	11 (2)	19 (11)	7 (2)	22 (8)	23 (20)	15 (7)	14 (3)	33 (32)
Number (%) female	7,643 (47)	479 (57)	135 (50)	7 (33)	25 (66)	78 (55)	84 (49)	75 (58)
Number (%) with liquid stool	15,543 (96)	787 (93)	250 (93)	18 (86)	35 (92)	140 (98)	166 (97)	129 (100)
Number (%) with bloody stool	1,174 (7)	213 (25)	42 (16)	2 (10)	8 (21)	6 (4)	11 (6)	5 (4)
Number (%) with vomiting	7,053 (44)	231 (27)	71 (26)	7 (33)	4 (11)	75 (53)	72 (42)	77 (60)
Number (%) who had taken antibiotics	607 (4)	42 (6)	8 (3)	0	0	5 (4)	3 (2)	1 (1)
Number (%) with severe dehydration	168 (1)	4 (1)	0	0	0	14 (10)	5 (3)	0
Number (%) with fever	5,164 (33)	279 (34)	89 (33)	8 (38)	7 (19)	24 (17)	52 (31)	25 (19)
Number (%) orally rehydrated	11,597 (72)	547 (65)	194 (72)	14 (67)	26 (68)	108 (76)	126 (74)	91 (71)
Number (%) intravenously rehydrated	2,338 (14)	77 (9)	12 (4)	2 (10)	2 (5)	49 (34)	23 (14)	33 (26)
Number (%) prescribed antibiotics	14,800 (97)	803 (98)	253 (98)	20 (100)	29 (100)	127 (98)	153 (90)	120 (93)
Number (%) referred to hospital	2,044 (13)	57 (7)	13 (5)	0	2 (5)	31 (22)	20 (12)	20 (16)

## Pre-publication history

The pre-publication history for this paper can be accessed here:



## References

[B1] Bern C, Martines J, de Zoysa I, Glass RI (1992). The magnitude of the global problem of diarrhoeal disease: a ten-year update. Bull World Health Organ.

[B2] Kosek M, Bern C, Guerrant RL (2003). The global burden of diarrhoeal disease, as estimated from studies published between 1992 and 2000. Bull World Health Organ.

[B3] Snyder JD, Merson MH (1982). The magnitude of the global problem of acute diarrhoeal disease: a review of active surveillance data. Bull World Health Organ.

[B4] Sunoto (1987). Diarrhoeal problems in Indonesia past, present and future. J Singapore Paediatr Soc.

[B5] Sunoto (1987). Diarrhoeal problems in South East Asia, 1986. Paediatr Indones.

[B6] Nazir M, Pardede N, Ismail R (1985). The incidence of diarrhoeal diseases and diarrhoeal diseases related mortality in rural swampy low-land area of south Sumatra, Indonesia. J Trop Pediatr.

[B7] Simanjuntak CH, Hardjining S, Hasibuan MA, Koiman I, Pujarwoto (1998). Gastrointestinal infections in Southeast Asia (V): ; Japan..

[B8] Lesmana M, Subekti D, Simanjuntak CH, Tjaniadi P, Campbell JR, Oyofo BA (2001). Vibrio parahaemolyticus associated with cholera-like diarrhea among patients in North Jakarta, Indonesia. Diagn Microbiol Infect Dis.

[B9] Oyofo BA, Subekti D, Tjaniadi P, Machpud N, Komalarini S, Setiawan B, Simanjuntak C, Punjabi N, Corwin AL, Wasfy M, Campbell JR, Lesmana M (2002). Enteropathogens associated with acute diarrhea in community and hospital patients in Jakarta, Indonesia. FEMS Immunol Med Microbiol.

[B10] Oyofo BA, Lesmana M, Subekti D, Tjaniadi P, Larasati W, Putri M, Simanjuntak CH, Punjabi NH, Santoso W, Sarumpaet S, Abdi M, Tjindi R, Ma'ani H, Sumardiati A, Handayani H, Campbell JR, Alexander WK, Beecham HJ, Corwin AL, Muzahar, Sukarma, Sriwati (2002). Surveillance of bacterial pathogens of diarrhea disease in Indonesia. Diagn Microbiol Infect Dis.

[B11] Richie E, Punjabi NH, Corwin A, Lesmana M, Rogayah I, Lebron C, Echeverria P, Simanjuntak CH (1997). Enterotoxigenic Escherichia coli diarrhea among young children in Jakarta, Indonesia. Am J Trop Med Hyg.

[B12] Lesmana M, Subekti DS, Tjaniadi P, Simanjuntak CH, Punjabi NH, Campbell JR, Oyofo BA (2002). Spectrum of vibrio species associated with acute diarrhea in North Jakarta, Indonesia. Diagn Microbiol Infect Dis.

[B13] Simanjuntak CH, Larasati W, Arjoso S, Putri M, Lesmana M, Oyofo BA, Sukri N, Nurdin D, Kusumaningrum RP, Punjabi NH, Subekti D, Djelantik S, Lubis A, Siregar H, Mas'ud B, Abdi M, Sumardiati A, Wibisana S, Setiawan B, Santoso W, Putra E, Sarumpaet S, Ma'ani H, Lebron C, Soeparmanto SA, Campbell JR, Corwin AL, Sukarma, Sriwati, Muzahar, Hendarwanto (2001). Cholera in Indonesia in 1993-1999. AmJ TropMed Hyg.

[B14] Indonesian-Government (2000). National Statistics Bureaucratic(BPS).

[B15] Richie EE, Punjabi NH, Sidharta YY, Peetosutan KK, Sukandar MM, Wasserman SS, Lesmana MM, Wangsasaputra FF, Pandam SS, Levine MM, O'Hanley PP, Cryz SJ, Simanjuntak CH (2000). Efficacy trial of single-dose live oral cholera vaccine CVD 103-HgR in North Jakarta, Indonesia, a cholera-endemic area. Vaccine.

[B16] Simanjuntak CH, O'Hanley P, Punjabi NH, Noriega F, Pazzaglia G, Dykstra P, Kay B, Budiarso A, Rifai AR, Suharyono (1993). Safety, immunogenicity, and transmissibility of single-dose live oral cholera vaccine strain CVD 103-HgR in 24- to 59-month-old Indonesian children. J Infect Dis.

[B17] Simanjuntak CH, Punjabi NH, Wangsasaputra F, Nurdin D, Pulungsih SP, Rofiq A, Santoso H, Pujarwoto H, Sjahrurachman A, Sudarmono P, von Seidlein L, Acosta C, Robertson SE, Ali M, Lee H, Park J, Deen JL, Agtini MD, Clemens JD (2004). Diarrhoea episodes and treatment-seeking behaviour in a slum area of North Jakarta, Indonesia. J Health Popul Nutr.

[B18] Vollaard AM, Ali S, van Asten HA, Widjaja S, Visser LG, Surjadi C, van Dissel JT (2004). Risk factors for typhoid and paratyphoid fever in Jakarta, Indonesia. Jama.

[B19] McLaughlin JC, Murray PR, Baron EJ, Pfaller MA, Tenover FC and Yolken RH (1995). Manual of Clinical Microbiology.

[B20] National-Committee-for-Clinical-Laboratory-Standards (2000). Performance standards for antimicrobial susceptibility testing: Tenth informational supplement M100-S10.

[B21] Perera BJ, Ganesan S, Jayarasa J, Ranaweera S (1999). The impact of breastfeeding practices on respiratory and diarrhoeal disease in infancy: a study from Sri Lanka. J Trop Pediatr.

[B22] Escobar GJ, Salazar E, Chuy M (1983). Beliefs regarding the etiology and treatment of infantile diarrhea in Lima, Peru. Soc Sci Med.

[B23] Nakao RM, Kennedy KI, Savina G (1992). Breastfeeding education and infant health in the rural Philippines. Ecol Food Nutr.

[B24] Kotloff KL, Winickoff JP, Ivanoff B, Clemens JD, Swerdlow DL, Sansonetti PJ, Adak GK, Levine MM (1999). Global burden of Shigella infections: implications for vaccine development and implementation of control strategies. Bull World Health Organ.

[B25] World-Health-Organization. (2000). Management of the child with a serious infection or severe malnutrition: Guidelines for care at the first referral level in developing countries (WHO/FCH/CAH/00.1).

[B26] Bhattacharya SK, Datta D, Bhattacharya MK, Garg S, Ramamurthy T, Manna B, Nair GB, Nag A, Moitra A (1992). Cholera in young children in an endemic area. Lancet.

[B27] Sack RB, Siddique AK, Longini IMJ, Nizam A, Yunus M, Islam MS, Morris JGJ, Ali A, Huq A, Nair GB, Qadri F, Faruque SM, Sack DA, Colwell RR (2003). A 4-year study of the epidemiology of Vibrio cholerae in four rural areas of Bangladesh. J Infect Dis.

[B28] Tuyet DT, Thiem VD, Von Seidlein L, Chowdhury A, Park E, Canh do G, Chien BT, Van Tung T, Naficy A, Rao MR, Ali M, Lee H, Sy TH, Nichibuchi M, Clemens J, Trach DD (2002). Clinical, epidemiological, and socioeconomic analysis of an outbreak of Vibrio parahaemolyticus in Khanh Hoa Province, Vietnam. J Infect Dis.

