# Correction: The burden of diarrhoea, shigellosis, and cholera in North Jakarta, Indonesia: findings from 24 months surveillance

**DOI:** 10.1186/1471-2334-7-1

**Published:** 2007-01-26

**Authors:** Magdarina D Agtini, Rooswanti Soeharno, Murad Lesmana, Narain H Punjabi, Cyrus Simanjuntak, Ferry Wangsasaputra, Dazwir Nurdin, Sri Pandam Pulungsih, Ainur Rofiq, Hari Santoso, H Pujarwoto, Agus Sjahrurachman, Pratiwi Sudarmono, Lorenz von Seidlein, Jacqueline L Deen, Mohammad Ali, Hyejon Lee, Deok Ryun Kim, Oakpil Han, Jin Kyung Park, Agus Suwandono, Ingerani, Buhari A Oyofo, James R Campbell, H James Beecham, Andrew L Corwin, John D Clemens

**Affiliations:** 1 National Institute of Health Research and Development, Jakarta Indonesia, Ministry of Health, Jakarta, Indonesia; 2 United States Navy Medical Research Unit 2, Jakarta, Indonesia; 3 Infectious Disease Hospital Sulianti Saroso, Ministry of Health, Jakarta, Indonesia; 4 Communicable Disease Control and Environmental Health, Ministry of Health, Jakarta, Indonesia; 5 Department of Microbiology, University of Indonesia, Jakarta, Indonesia; 6 International Vaccine Institute, Seoul, Korea; 7 National Institute of Child Health and Human Development, Bethesda, Maryland, USA

## Abstract

This is a correction of an earlier published article.

## Text

After the publication of this work[1], we became aware that the our description of the methods used to identify *Shigella *spp. did not describe the identification of a subset of *Shigella *spp. which were untypeable with commercial sera. Additional typing was conducted by Dr. Kaisar A. Talukder and coworkers (Laboratory Sciences Division, ICDDR, B: Centre for Health and Population Research, GPO Box-128, Dhaka-1000, Bangladesh. Email:kaisar@icddrb.org.)

## Brief description of the methods used for serotyping the untypeable *S. flexneri *strains using MASF

All *Shigella *strains which agglutinated with commercial polyvalent anti-*S. flexneri *serum (Denka Seiken, Tokyo, Japan) but were untypeable using commercial serotype specific antisera were examined using a panel of monoclonal antibodies against *S. flexneri *(MASF B) according to the typing scheme shown in Table 1 using monoclonal antibodies from one supplier (Reagensia AB, Stockholm, Sweden) with the exception of MASF 1c which was provided by Dr. N. I. A. Carlin (SBL, Sweden) [2-5]. Untypeable *S. flexneri *strains which agglutinated strongly with polyclonal MASF B and type antigen factor IV-2 but not with group factor Y-5 or 6 thereby excluding their characterization into serotype 4a or 4b were designated as serotype 4 if they agglutinated with a new antigenic determinant E1037 (IV-1). Untypeable strains which agglutinated with antibodies against the provisional antigen, MASF IV-1 specific for the new antigenic determinant E1037, were designated as 4X. Untypeable strains which agglutinated only with MASF Ic were designated as 1c.

**Table 1:** *S. flexneri *serotyping criteria using MASF antibodies

Serotype	Type antigen specific					MASF B	Group antigen specific			Provisional antigen specific	
	
	1 (1:-)^a^	11 (11:-)	IV-2 (IV:-)	V (V:-)	VI (VI:-)	B (-:1)	Y-5 (-:3.4)	6 (-:6)	7.8 (-:7.8)	IV-1 (-:E1037)	1c
1a	+					+	+				
1b	+					+		+			
**1c**						**+**					**+**
2a		+				+	+				
2b		+				+			+		
3a						+		+	+		
3b						+	+	+			
3c						+		+			
**4:-**			**+**			**+**				**+**	
4a			+			+	+			+/-	
4b			+			+		+			
5a				+		+	+				
5b				+		+			+		
6					+	+				+/-	
X						+			+	+/-	
Y						+	+			+/-	
**4X**						**+**				**+**	

^a ^Shown in parentheses is the antigen recognized.

+ Positive reaction

## Pre-publication history

The pre-publication history for this paper can be accessed here:



## References

[B1] Agtini MD, Soeharno R, Lesmana M, Punjabi NH, Simanjuntak C, Wangsasaputra F, Nurdin D, Pulungsih SP, Rofiq A, Santoso H, Pujarwoto H, Sjahrurachman A, Sudarmono P, von Seidlein L, Deen JL, Ali M, Lee H, Kim DR, Han O, Park JK, Suwandono A, Oyofo BA, Campbell JR, Beecham HJ, Corwin AL, Clemens JD, Ingerani (2005). The burden of diarrhoea, shigellosis, and cholera in North Jakarta, Indonesia: findings from 24 months surveillance. BMC Infect Dis.

[B2] Carlin NI, Rahman M, Sack DA, Zaman A, Kay B, Lindberg AA (1989). Use of monoclonal antibodies to type Shigella flexneri in Bangladesh. J Clin Microbiol.

[B3] Talukder KA, Dutta DK, Safa A, Ansaruzzaman M, Hassan F, Alam K, Islam KM, Carlin NI, Nair GB, Sack DA (2001). Altering trends in the dominance of Shigella flexneri serotypes and emergence of serologically atypical S. flexneri strains in Dhaka, Bangladesh. J Clin Microbiol.

[B4] Talukder KA, Islam MA, Dutta DK, Hassan F, Safa A, Nair GB, Sack DA (2002). Phenotypic and genotypic characterization of serologically atypical strains of Shigella flexneri type 4 isolated in Dhaka, Bangladesh. J Clin Microbiol.

[B5] Talukder KA, Islam Z, Islam MA, Dutta DK, Safa A, Ansaruzzaman M, Faruque AS, Shahed SN, Nair GB, Sack DA (2003). Phenotypic and genotypic characterization of provisional serotype Shigella flexneri 1c and clonal relationships with 1a and 1b strains isolated in Bangladesh. J Clin Microbiol.

